# Trace amine associated receptor 1: predicted effects of single nucleotide variants on structure-function in geographically diverse populations

**DOI:** 10.1186/s40246-024-00620-w

**Published:** 2024-06-11

**Authors:** Britto Shajan, Shashikanth Marri, Tarun Bastiampillai, Karen J. Gregory, Shane D. Hellyer, Pramod C. Nair

**Affiliations:** 1 Discipline of Clinical Pharmacology, College of Medicine and Public Health, Flinders University, Adelaide, SA Australia; 2 Flinders Health and Medical Research Institute (FHMRI) College of Medicine and Public Health, Flinders University, Adelaide, SA Australia; 3 Department of Psychiatry, Monash University, Parkville, Melbourne, VIC Australia; 4 Discipline of Psychiatry, College of Medicine and Public Health, Flinders University, Adelaide, SA Australia; 5 Drug Discovery Biology, Monash Institute of Pharmaceutical Sciences, Monash University, 381 Royal Parade, Melbourne, VIC 3052 Australia; 6 ARC Centre for Cryo-electron Microscopy of Membrane Proteins, Monash Institute of Pharmaceutical Sciences, Monash University, Parkville, VIC 3052 Australia; 7 South Australian Health and Medical Research Institute, University of Adelaide, Adelaide, South Australia Australia; 8 Discipline of Medicine, Adelaide Medical School, University of Adelaide, Adelaide, South Australia Australia

**Keywords:** Genetic variants, Trace amines, Neuropsychiatric disorders, Agonists, Neurotransmitters, Ulotaront, Ralmitaront, Amphetamines, Demographic, Aminergic receptors

## Abstract

Trace Amine Associated Receptor 1 (TAAR1) is a novel pharmaceutical target under investigation for the treatment of several neuropsychiatric conditions. TAAR1 single nucleotide variants (SNV) have been found in patients with schizophrenia and metabolic disorders. However, the frequency of variants in geographically diverse populations and the functional effects of such variants are unknown. In this study, we aimed to characterise the distribution of TAAR1 SNVs in five different WHO regions using the Database of Genotypes and Phenotypes (dbGaP) and conducted a critical computational analysis using available TAAR1 structural data to identify SNVs affecting ligand binding and/or functional regions. Our analysis shows 19 orthosteric, 9 signalling and 16 micro-switch SNVs hypothesised to critically influence the agonist induced TAAR1 activation. These SNVs may non-proportionally influence populations from discrete regions and differentially influence the activity of TAAR1-targeting therapeutics in genetically and geographically diverse populations. Notably, our dataset presented with orthosteric SNVs D103^3.32^N (found only in the South-East Asian Region and Western Pacific Region) and T194^5.42^A (found only in South-East Asian Region), and 2 signalling SNVs (V125^3.54^A/T252^6.36^A, found in African Region and commonly, respectively), all of which have previously demonstrated to influence ligand induced functions of TAAR1. Furthermore, bioinformatics analysis using SIFT4G, MutationTaster 2, PROVEAN and MutationAssessor predicted all 16 micro-switch SNVs are damaging and may further influence the agonist activation of TAAR1, thereby possibly impacting upon clinical outcomes. Understanding the genetic basis of TAAR1 function and the impact of common mutations within clinical populations is important for the safe and effective utilisation of novel and existing pharmacotherapies.

**Supplementary Information:**

The online version contains supplementary material available at 10.1186/s40246-024-00620-w.

## Introduction

The endogenous trace amines (TAs) are small molecules that selectively activate a group of G protein-coupled receptors (GPCRs) called trace amine associated receptors (TAARs) [1, 2]. Human TAARs are encoded by an intron-less cluster of *Taar* genes located on chromosome 6q23.2, with six genes encoding for functional TAARs (TAAR1, 2, 5, 6, 8 and 9) [3]. TAARs are distantly related to monoamine neurotransmitter GPCRs and share close structural similarities with the broader class A GPCR family, featuring a membrane spanning hepta-helical transmembrane domain (TM) and three intracellular and extracellular loops (ICL1-3 and ECL1-3, respectively) [1]. While most TAARs function primarily as olfactory receptors, with low CNS expression, TAAR1 is highly expressed in the CNS, particularly in monoaminergic nuclei [1, 2]. Furthermore, TAAR1 expression is also reported in peripheral systems including the gastrointestinal tract, heart, thyroid, and immune systems [4]. When inactive, TAAR1 is proposed to be localised on the endoplasmic reticulum and mitochondrial membranes [4]. However, TAAR1 activation via trace amine binding and/or heterodimerisation with other receptors triggers translocation to the plasma membrane and the production of cellular responses, primarily by altering intracellular cyclic-adenosine monophosphate (cAMP) levels [5]. Through these actions, TAAR1 plays important roles in the modulation of monoaminergic and glutamatergic neurotransmission, hormone secretion and glucose metabolism, regulating vital processes involved with cognition, mood, and metabolism [2, 3]. As such, TAAR1 has arisen as the major focus of TAAR research due to its therapeutic potential in treating CNS and metabolic disorders [2].

The critical role of TAAR1 in modulating monoaminergic neurotransmission is exemplified in TAAR1 knockout mice, which display hypersensitivity to amphetamine and elevated release of monoaminergic neurotransmitters [5, 6]. Further, receptor stimulation with partial TAAR1 agonists significantly improved psychological distress, cognitive functioning, sleep patterns, and produced anti-diabetic effects in preclinical models of schizophrenia and diabetes [6–9]. Many structurally distinct TAAR1 full and partial agonists have since been developed, with several entering clinical trials for schizophrenia, anxiety, Parkinson’s L-Dopa related psychosis, sleep disorder and diabetes [10]. Of note is ulotaront (SEP-363856), a dual TAAR1/5-HT1A agonist, which received FDA Breakthrough Therapy status for schizophrenia treatment in 2019 [11, 12]. However, despite promising preclinical and early clinical results, ulotaront failed to hit primary endpoints in two recent Phase 3 trials for schizophrenia (ClinicalTrials.gov Identifiers: NCT04072354, NCT04092686) [13, 14]. Initial reports suggested a high placebo effect masking the therapeutic effects of ulotaront as the primary reason for the clinical failures [15]. However, it remains unknown exactly what factors play a role in a lack of ulotaront efficacy in patient populations. As Phase 2 clinical trials for ulotaront are currently underway as a treatment for generalised anxiety disorder (ClinicalTrials.gov Identifier: NCT05729373) and as adjunctive therapy for major depressive disorder (ClinicalTrials.gov Identifier: NCT05593029), understanding the reasons for this failure is of critical importance for the future clinical success of both ulotaront and other TAAR1 clinical candidates [16, 17].

Genetic mutations are of particular interest in GPCR drug discovery due to their disease-causing effects and potential to interfere with drug activity by disrupting drug metabolism, protein-ligand interactions, and receptor function [18–21]. A partial TAAR1 agonist, Ro5263397, failed Phase 1 clinical trials due to splicing mutations in the drug metabolism enzyme uridine 5′-diphosphate-glucuronosyltransferase 2B10, which increased exposure to parent compound by more than 100-fold [22]. The causative single nucleotide variant (SNV) mutation was particularly pervasive in African regions, indicating geographical heterogeneity in susceptibility to altered drug effects [22]. The TAAR1 gene cluster has also been identified via linkage analysis as a locus of susceptibility for schizophrenia, with clinical and experimental studies showing correlation between TAAR1 SNVs and patients with impaired glucose homeostasis and schizophrenia in geographically distinct populations [19, 23, 24]. Indeed, numerous SNVs identified in human TAAR1 alter expression and functional properties, with previous studies relying on mutagenesis and in vitro characterisation to test the functional effects of TAAR1 SNVs [19, 23, 25, 26]. Furthermore, multiple high resolution cryo-electron microscopy (cryo-EM) TAAR1 structures bound to both natural TAs and synthetic agonists have recently been solved [27–31]. Such advances pave the way for structure-based approaches to studying TAAR1 ligand interactions and structure-function, which until recently have relied on homology models [32]. Computational approaches using experimentally determined structures represent a powerful method for rapidly screening the effects of TAAR1 SNVs that potentially impact drug action in genetically and geographically diverse clinical populations.

The purpose of this study was to leverage recently published experimental structures of TAAR1 to identify the putative effects of SNVs on TAAR1 structure-function using bioinformatic approaches. The Database of Genotypes and Phenotypes (dbGaP) was used to identify and characterise putative detrimental SNVs in populations belonging to five diverse geographical regions. With the aim of predicting the impact of these SNVs on TAAR1 function, we have identified 19 SNVs at the orthosteric binding sites, 9 at signalling domains and 16 at micro-switch domains. Additionally, in silico functional prediction algorithms predicted all 16 micro-switch SNVs are damaging to TAAR1 function. We found several crucial mutations affecting conserved regions that are geographically non-uniform in their distribution. The results of our study provide a basis for further in silico and in vitro studies that may provide deeper insights and experimental validation for TAAR1 SNV functional effects.

## Methods

### SNV data from NCBI dbGaP repository

All SNV data were collected from the National Center for Biotechnology Information (NCBI) dbGaP database (2023) [33]. The Reference SNP cluster ID (rsID) were compiled based on five of WHO classification of regions, African Region (AR), South-East Asian Region (SEAR), Region of the Americas (ROA), European Region (ER) and Western Pacific Region (WPR). Regions containing pan-ethnic groups, such as Region of the Americas, were classified based on their genetic architecture [34].

### Prediction of SNV functional effects using in silico tools

To analyse the effect of specific TAAR1 SNVs, in silico algorithms were employed to predict tolerability. The tools were accessed through dbNSFP (version 4.4a), a repository containing a range of functional prediction algorithms [35]. To prevent ambiguity in relation to cut-off scores, tools yielding a binary output as damaging (D) or Tolerable (T) were selected. Furthermore, tools that utilised High, Medium, and Low scoring formats were considered. All High outputs were characterised as damaging, while Medium and Low outputs were characterised as tolerable. Four algorithms satisfied these inclusion criteria. (1) Sorting Intolerant from Tolerant (SIFT4G v2.4), which predicts the likelihood of a mutation to cause functional damage using sequence homology, assumes that evolutionarily conserved regions are essential for protein function and therefore any missense substitution is generally considered intolerable [36]. (2) Protein Variation Effect Analyzer (PROVEAN v1.1) utilises sequence homology to estimate the tolerability of a mutation by computing the change in delta alignment score using the BLOSUM62 substitution matrix [37]. (3) Mutation Assessor (v3.0) utilises sequences from homologous protein families and sub-families within and between species, to calculate an impact probability score [38]. (4) MutationTaster 2 (v2015) utilises machine learning approaches and is trained using > 390,000 disease variants and > 6 million tolerable polymorphisms. MutationTaster 2 uses Bayes classifier algorithm to calculate the probability of a mutation being damaging or tolerable [39].

### Identification of TAAR1 SNVs at ligand binding and other functional domains through structural mapping

The snake plot of human TAAR1 (TA1 human) was acquired from GPCRdb [40]. To identify SNVs at the ligand binding and other functional domains of TAAR1, current literature on human TAAR1 experimental structures were utilised. The complexes of TAAR1-ligand (8W8A, 8W89, 8W88, 8W87, 8JSO, 8JLN, 8JLO, 8JLP, 8JLQ, 8JLR, 8WC8, 8WCA and 8UHB) were accessed using the RCSB PDB database [27–30]. Search term “TAAR1” was provided to make the initial query and the output was refined using “Homo sapiens” under ‘Scientific Name of Source Organism’. The resulting structures and primary literature were reviewed to identify residues involved in ligand activated mechanisms of TAAR1.

### Generating plots using bioinformatic tools and online databases

The position of SNVs within the TAAR1 structure were sorted based on the geographical distribution and a 5-way Venn diagram was generated using the Venn package through R studio version 2023.06. In addition, the geographical distribution of each SNV were segregated and illustrated using river plots, generated using ggalluvial package in RStudio version 2023.06. ChimeraX (version 1.7.1) and OpenEye Scientific VIDA (version 5.0.5.3) were utilised for visualisation and annotations of 3D structures (41–43).

## Results

### Allelic heterogeneity and geographical distribution of human TAAR1 SNVs

**Fig. 1:**
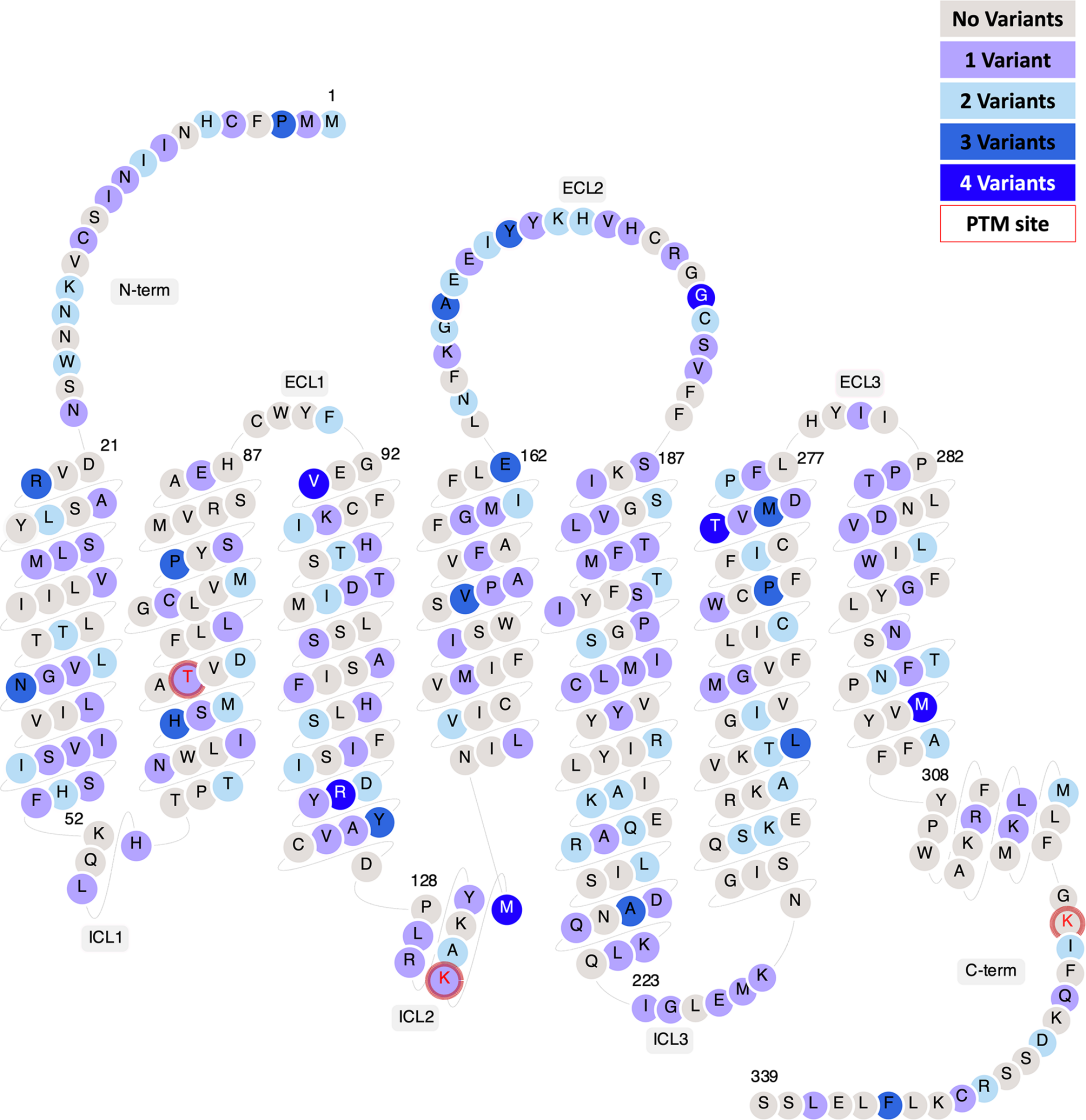
Snake plot of human TAAR1 demonstrating allelic heterogeneity observed in this dataset. The data was accrued from NCBI dbGaP consisting of 290 TAAR1 SNVs. The allelic heterogeneity was characterised using the TAAR1 snake plot, accessed from GPCRdb (TA1 human structure). As shown, up to four variants were originated from six individual residues. Three predicted post-translational modification sites (PTM sites) have been identified (T67^2.48^ (phosphorylation), K134^ICL2^ (methylation) and K322^C − term^ (acetylation)) using GPCRdb, with two residues affected by SNVs (T67^2.48^ and K134^ICL2^).

A list of rare missense TAAR1 SNVs was collated from the NCBI dbGaP database. To identify the geographical distribution of each SNV, the reference SNP cluster ID (rsID) were classified based on five WHO regions, African Region (AR), South-East Asian Region (SEAR), Region of the Americas (ROA), European Region (ER), and Western Pacific Region (WPR). A total of 290 individual TAAR1 SNVs were identified, all of which are segregated at varying capacity, covering all domains of TAAR1 including TM, ICL, ECL, N-terminal extension and C-terminal tail. To visualise the position of individual SNVs and identify allelic heterogeneity at each residue, SNVs were mapped onto a TAAR1 snake plot from GPCRdb (Fig. 1) [40]. As highlighted, the current dataset shows 108 residues with a single SNV, which constitutes approximately 37% of this dataset. The remaining 63% are segregated as residues with double, triple, and quadruple allelic heterogeneity. Notably, the highest heterogeneity (four variants) was displayed at six positions; V94^3.23^ (Superscript is position according to Ballesteros & Weinstein numbering system, which indicates helix position of residue relative to the most conserved amino acid within a TM [44]), R121^3.50^ and M135^ICL2^, G181^ECL2^, T271^6.55^ and M302^7.51^. In addition, SNVs in two post translational modification sites (Predicted PTM site, identified using GPCRdb) were identified, T67^2.48^, K134^ICL2^ [40]. Overall, this dataset presents with SNVs covering approximately 55% (186 residues) of human TAAR1.

**Fig. 2:**
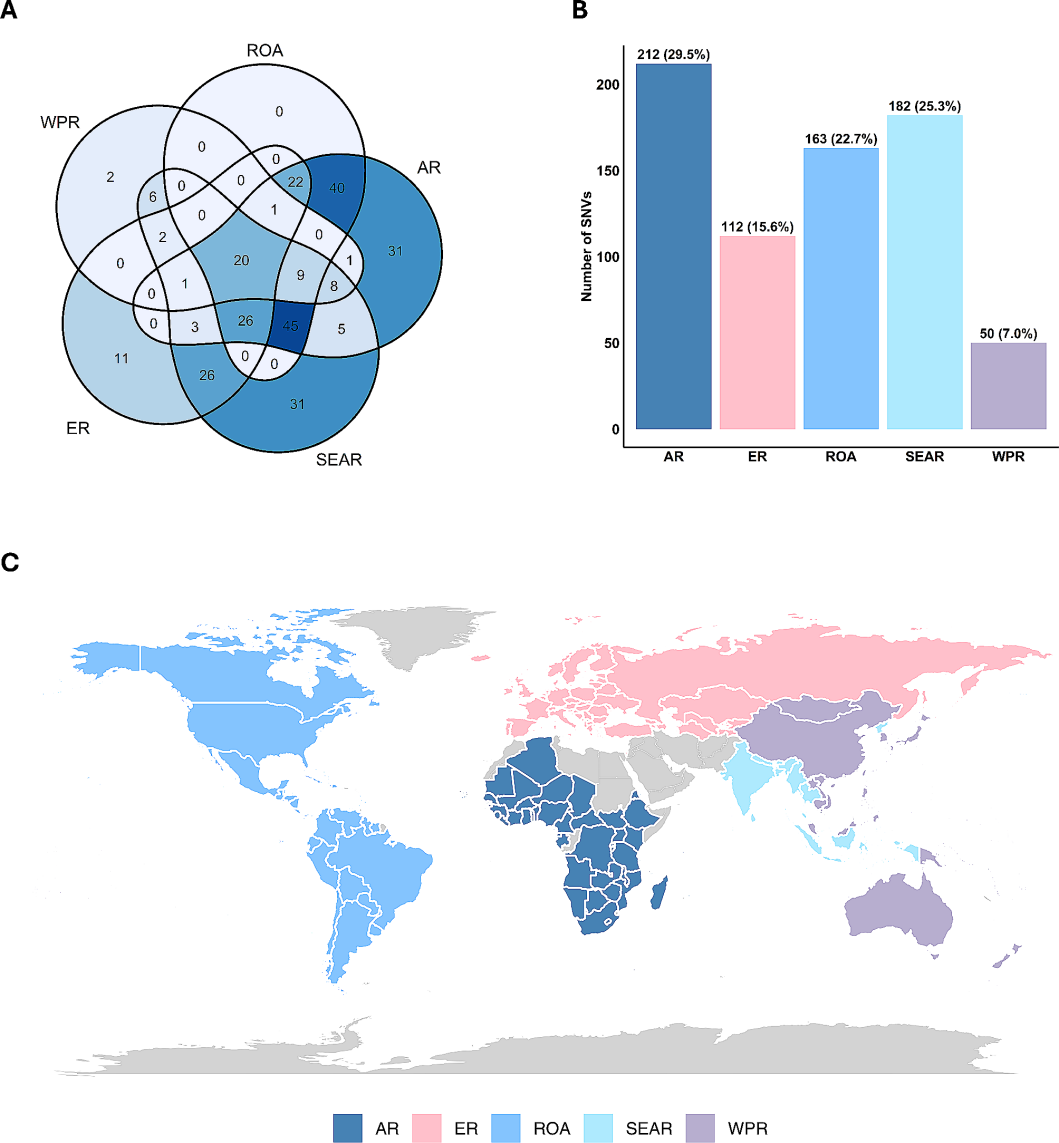
Distribution of TAAR1 SNVs across different demographic regions. The data was accrued from NCBI dbGaP and characterised into five WHO regions of classifications; WPR, AR, ROA, SEAR, and ER. (**A**) Venn analysis showing the distribution of SNVs across five regions. The analysis was conducted using the ggVenndiagram package in R studio. (**B**) Burden of TAAR1 SNV in each region. All SNVs, including unique and shared variants found in each demographic region were tallied. (**C**) World map showing each WHO regions of classification. Eastern Mediterranean Region and Antarctica (grey colour) were not considered in this study due to very low SNV prevalence. The bar graph and World map was generated in R studio using ggplot and maps packages, respectively

To characterise the geographical distribution and consequently define shared and unshared SNVs, a Venn analysis was performed. As shown in Figs. 2A and C, 20 SNVs are shared across all five geographical regions (referred to as “common SNVs” henceforth) and a total of 75 SNVs are unique. Notably, unique SNVs were most pervasive in SEAR and AR (31 unique SNVs in each) and no unique SNVs were identified in the ROA. To elucidate the prevalence of TAAR1 SNVs regionally, we defined the burden of TAAR1 SNVs by tallying all the unique and shared SNVs present in each region (Fig. 2B). In this dataset, the total burden of TAAR1 mutations were the highest in AR (*n* = 212) and lowest in the WPR (*n* = 50).

To best illustrate our overall data, a river plot was utilised (Supplementary Figs. 1–4). Briefly, all plots were composed of two complementary horizontal axes, top axis with the WHO region and bottom axis with the SNVs. Both axes are bridged by up to five “streams”, each representing a WHO region. In this case, the stream can be seen to originate from the axis of WHO region and links to a single/multiple SNVs that is in association with the region. The stream also can be interpreted to originate from the axis of SNVs and linking to a WHO region/s to precisely locate the region/s in association with SNV. Thus, the relative axial area, i.e. the size of each box a given SNV, or a WHO region occupies, represents the sum of associations with its complementary variable. In addition, shared SNVs are presented using the grey shade and unique SNVs are presented with varying colour shades corresponding to the WHO region (e.g. V288^7.37^A in AR with sky blue).

### SNVs at the ligand binding regions of human TAAR1

The potential impact of identified SNVs on important aspects TAAR1 function, such as agonist binding and signalling remain unexplored. Hence, our analysis focused on studying the SNVs present in the TAAR1 agonist binding and signalling domains. With the emergence of various agonist bound TAAR1 cryo-EM structures, residues critical for differential agonist binding and signalling are beginning to unfold [27–30]. Considering this, we performed identical structural mapping analysis using the available cryo-EM structures to map the distribution of SNVs across TAAR1 ligand binding pockets. This was conducted in conjunction with the geographical data to analyse and define the influence of SNVs present in each region.

**Fig. 3:**
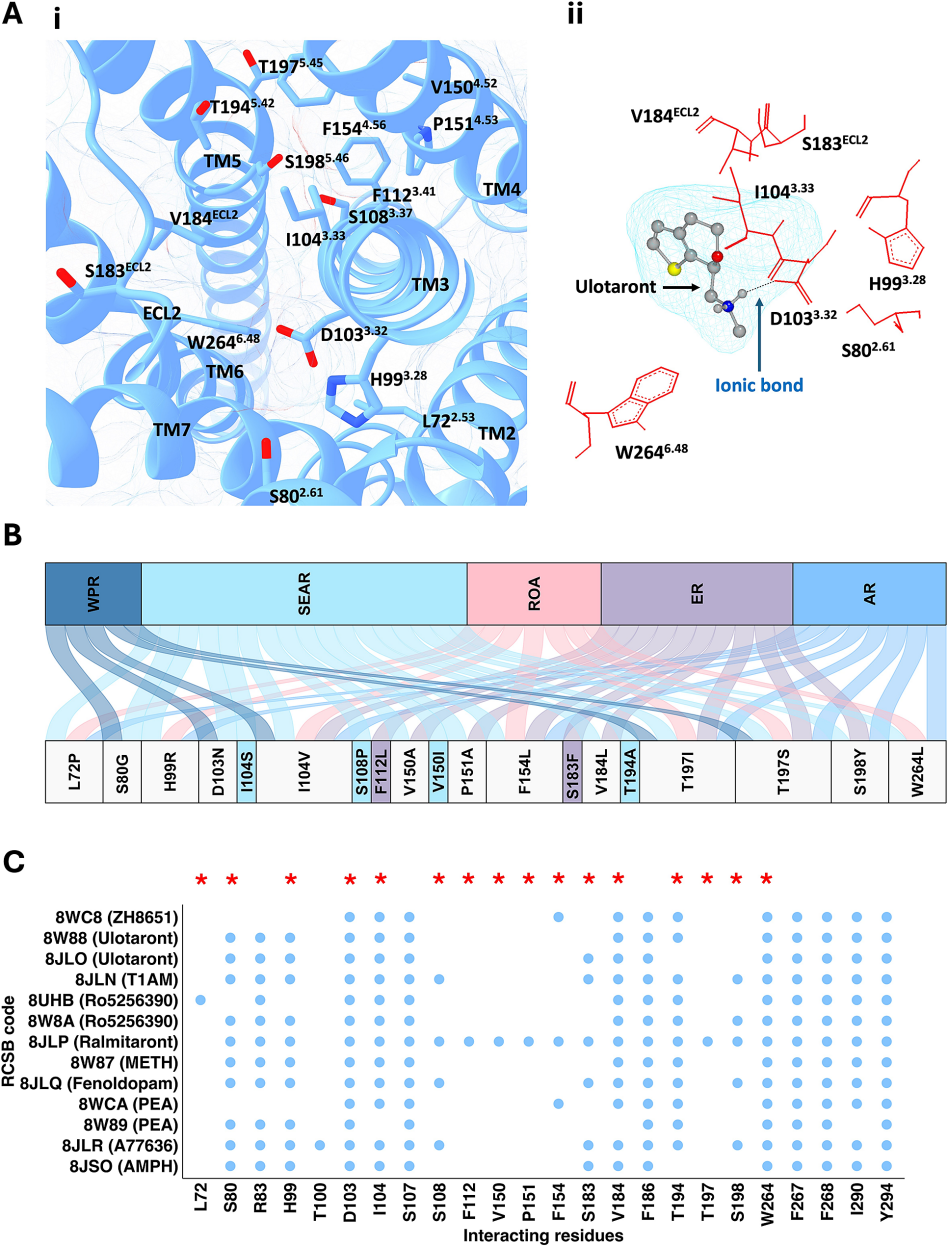
SNVs affecting residues involved in ligand binding, defined as orthosteric SNVs. Amino acid residues involved in ligand binding were curated using the human ligand-TAAR1 complexes deposited in RSCB, including 8W8A, 8W89, 8W88, 8W87, 8JSO, 8JLN, 8JLO, 8JLP, 8JLQ, 8JLR, 8JSO, 8WC8, 8WCA and 8UHB (ligand name is displayed in parenthesis). Primary SNV data was accrued from NCBI dbGaP and mapped on the ulotaront-TAAR1-Gαs coupled cryo-EM complex (RCSB: 8JLO). (**A**) Ulotaront-TAAR1-Gαs coupled cryo-EM complex (RCSB: 8JLO) (**i**) residues involved in synthetic and endogenous agonist binding affected by SNVs (shown in sticks), (**ii**) complexed ulotaront at the binding site with WT residues affected by SNVs, including D103^3.32^ which establishes a critical ionic bond required for ligand recognition. (**B**) River plot demonstrating the demographic distribution of orthosteric SNVs. Top panel displays the proportional burden of orthosteric SNVs in WPR, AR, ROA, SEAR and ER. Bottom panels show the list of orthosteric SNVs. The ribbons link each region with associated SNVs. Unique SNVs are colour matched with respective WHO region, and shared SNVs are coloured using light grey. (**C**) List of residues found to interact with complexed ligands. The river plot and scatter plot were generated using the ggalluvial package and ggplot of R studio, respectively. 3D structure depictions were created using ChimeraX and OpenEye Scientific VIDA. *Indicates presence of mutation/s. PEA (β-phenethylamine), METH (methamphetamine), AMPH (amphetamine) and T1AM (3-Iodothyronamine)

‘Orthosteric SNVs’ are defined as SNVs at residues surrounding the binding site of various TAAR1 agonists, identified from experimentally determined TAAR1 structures (Fig. 3) including RCSB entries 8W8A, 8W89, 8W88, 8W87, 8JSO, 8JLN, 8JLO, 8JLP, 8JLQ, 8JLR, 8WC8, 8WCA and 8UHB. A total of 19 orthosteric SNVs, affecting 16 residues were identified (Fig. 3A, supplementary Table 1). Three common orthosteric SNVs were identified at two positions, I104^3.33^ (variant; V104^3.33^) and T197^5.45^ (variants; I197^5.45^, S197^5.45^), which are crucial for the binding of several exogenous and endogenous TAAR1 agonists (Fig. 3B, C). Specifically, residue I104^3.33^ forms interactions with 3-iodothyronamine (T1AM), amphetamine and other synthetic agonists, while residue T197^5.45^ interacts with ralmitaront (Fig. 3C) [27–30]. As shown in Fig. 3B, six unique orthosteric SNVs were found, including I104^3.33^S, S108^3.37^P, F112^3.41^L, V150^4.52^I, S183^ECL2^F and T194^5.42^A. All residues form key interactions with ralmitaront, with residue specific interactions with other agonists such as T1AM (I104^3.33^, S108^3.37^, S183^ECL2^, T194^5.42^), ulotaront (I104^3.33^, S183^ECL2^, and T194^5.42^), amphetamine (I104^3.33^ and S183^ECL2^), methamphetamine (I104^3.33^ and T194^5.42^), β-phenylethylamine (PEA) (I104^3.33^ and T194^5.42^) and Ro5256390 (I104^3.33^ and T194^5.42^) [27–30] as illustrated in Fig. 3C. Apart from F112^3.41^L and S183^ECL2^F (both found in ER), all unique orthosteric SNVs belong to SEAR. Notably, shared orthosteric SNVs such as S80^2.61^G (WPR/SEAR), H99^3.28^R (ROA and others, Fig. 3B) and V184^ECL2^L (SEAR/ER) may influence the binding of endogenous compounds PEA and T1AM [27]. Furthermore, residues D103^3.32^ and W264^6.48^ interact with all TAAR1 agonists tested thus far (Fig. 3C), with a single shared SNV at each residue: D103^3.32^N (WPR and SEAR) and W264^6.48^L (AR, SEAR, and ROA) as illustrated in Fig. 3B [27–30].

### SNVs at the micro-switch domains of human TAAR1

SNVs found at the highly conserved micro-switch domains may influence TAAR1 functionality. Micro-switches are structural motifs conserved across the Class A GPCR superfamily that link ligand binding with conformational changes associated with G protein coupling and receptor activation [29]. From the cryo-EM data, it was evident TAAR1 has the typical micro-switches for the classical class A GPCR helical rearrangement [27–30]. These micro-switches include the DRY (D120^3.49^, R121^3.50^ and Y122^3.51^), PIF (P202^5.50^, I111^3.40^ and F260^6.44^), CWxP (C263^6.47^, W264^6.48^, C265^6.49^ and P266^6.50^), and NPxxY motifs (N300^7.49^, P301^7.50^, M302^7.51^, V303^7.52^ and Y304^7.53^).

**Fig. 4:**
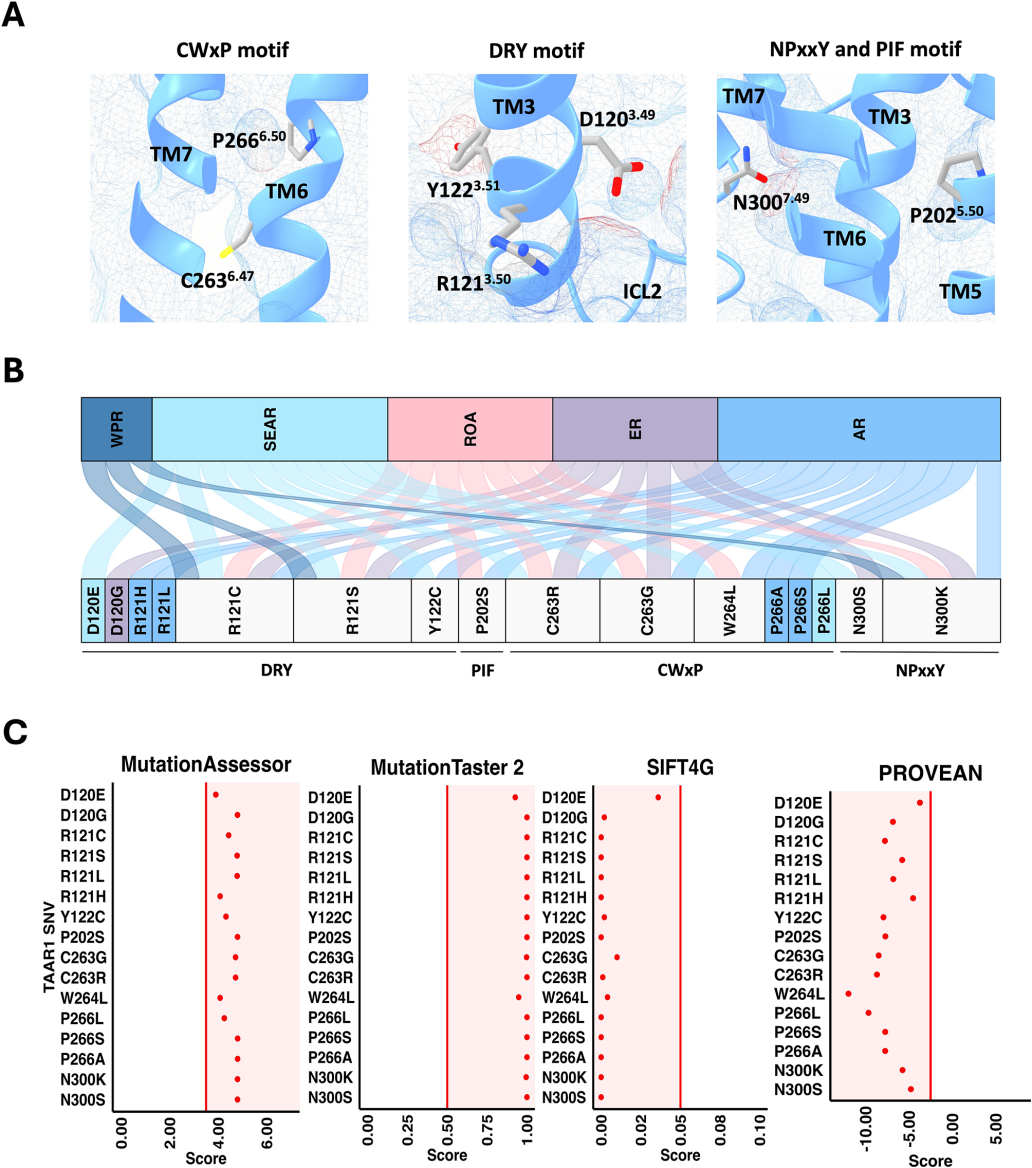
Demographic distribution and predicted effects of class A micro-switch SNVs. Primary SNV data was accrued from NCBI dbGaP and mapped on the ulotaront-TAAR1-Gαs cryo-EM complex (RCSB: 8JLO). (**A**) Micro-switch residues containing SNVs, shown in sticks; residues of CWxP motif with SNVs (W264^6.48^ not shown), residues of DRY motif with SNVs residues of NPxxY and PIF motif with SNVs (**B**) River plot displaying demographic distribution of microswitch SNVs. The bottom panel show the list of SNVs. The ribbons link each region with associated SNVs. Unique SNVs are colour matched with respective WHO region, and shared SNVs are coloured using light grey. (**C**) Prediction of putative effects of micro-switch SNVs using SIFT4G, MutationTaster 2, PROVEAN and MutationAssessor. The tools were accessed using dbNSFP database. Variants were called damaging upon meeting the following threshold scores (denoted by the red vertical line): <0.05 (SIFT4G), > 0.5 (MutationTaster 2), < -2.5 (PROVEAN) and > 3.5 (MutationAssesor)

Using structural mapping analysis, we classified SNVs within the four micro-switch domains as ‘micro-switch SNVs’ (Fig. 4A, Supplementary Table 2). A total of 16 micro-switch SNVs were identified, located within the DRY (seven SNVs), CWxP (six SNVs), PIF (one SNV) and NPxxY (two SNVs) motifs (Fig. 4B). When considering geographical distribution, three common micro-switch SNV were identified: R121^3.50^C/S affecting the DRY and N300^7.49^K affecting NPxxY motif. Furthermore, a total of seven unique micro-switch SNVs were identified; four in AR, two in SEAR and one in the ER as shown in Fig. 4B. Shared variants in micro-switch regions were also identified, one of each affecting DRY, PIF, NPxxY and three affecting CWxP (Fig. 4B).

Although the importance of the micro-switch regions in class A receptor rearrangement and activation are well known, the functional implications of SNVs in these motifs on TAAR1 activity remains largely unknown. Notably, putative deleterious effects of R121^3.50^C/L, C263^6.47^G, W264^6.48^L, P266^6.50^S/A and N300^7.49^S/K were previously described by GPCRdb using SIFT and PolyPhen algorithms. Therefore, to characterise the putative effects of TAAR1 micro-switch SNVs, four different *in silico* algorithms were employed to predict the effect of variants as either tolerable or damaging; SIFT4G, PROVEAN, Mutation Assessor and MutationTaster 2. All micro-switch SNVs were predicted to be damaging by all four selected algorithms (Fig. 4C).

### SNVs affecting the G protein-coupling residues of human TAAR1

**Fig. 5:**
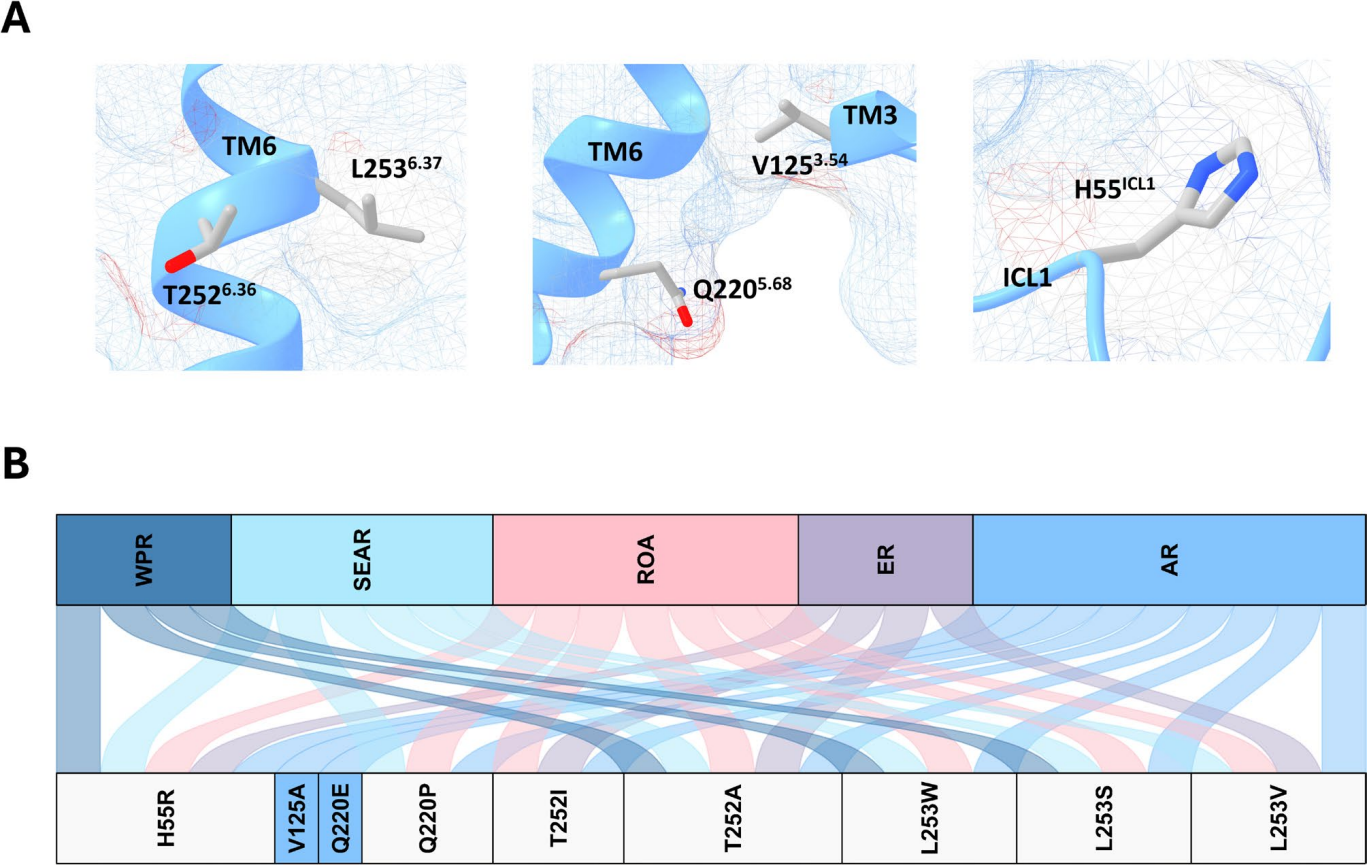
SNVs identified at the G-protein coupling interface of TAAR1, referred to as signalling SNVs. Primary SNV data was accrued from NCBI dbGaP and current cryo-EM literature was utilised to map signalling SNVs. (**A**) SNVs identified at residues demonstrated to be critical for different TAAR1 signalling pathways, shown in sticks. (**B**) River plot displaying demographic distribution of signalling SNVs. Unique SNVs are colour matched with respective WHO region, and shared SNVs are coloured using light grey. Alanine variants present in this subset has been functionally validated and shown to disrupt G-protein activity

Cryo-EM structures of TAAR1 in complex with structurally diverse ligands have revealed TAAR1 agonists engender divergent G protein coupling and downstream signalling outcomes, with mutagenesis revealing residues involved in this preferential G protein activation [27–30]. As such, we characterised 9 SNVs at five positions within TAAR1 identified as critical for divergent G protein activation and signalling transduction (referred to as signalling SNVs**)** (Fig. 5A, B). Two common signalling SNVs were identified, one affecting a residue important for TAAR1-G_αs_ subunit activity (H55^ICL1^R), and T252^6.36^A critical TAAR1-G_αi_ activity. Two unique SNVs were present in the AR; one affecting residue critical for TAAR1-G_αs_ activity (Q220^5.68^E), and other residue (V125^3.54^A), implicated with creating selective bias for G_αi_ over G_αs_ coupling [29]. Additional signalling SNVs were shared across multiple regions and may influence TAAR1-G_α_-protein interactions based on sites from previous experimental studies, as summarised in Fig. 5B [29].

## Discussion

A greater understanding of the impact of SNVs is warranted in light of emerging studies connecting TAAR1 SNVs with neuropsychiatric disorders [19, 23–25]. Here, demographic distribution and atomic insights of human TAAR1 experimental structures were utilised to define and predict effects of selected TAAR1 missense SNVs. Our findings showed a large degree of allelic heterogeneity, presence of multi-regional SNVs and implicated AR with highest overall count of SNVs. Our structural mapping analysis utilising cryo-EM structural insights isolated the primary data set consisting of over 40 SNVs. Within the dataset were 16 SNVs at four different micro-switch regions, with putative damaging effects predicted by four *in silico* algorithms. Here, AR had the highest number of micro-switch SNVs. In addition, 19 orthosteric SNVs, and 9 signalling SNVs were noted, with highest count was in SEAR and AR, respectively.

Structure-based predictions and bioinformatics approaches have been used to predict the functional effects of SNVs on multiple pharmacological targets [19, 45–48]. With the emergence of various agonist bound human TAAR1 cryo-EM structures, the binding residues for several TAAR1 agonists have been characterised [27–30]. The TAAR1 cryo-EM structures in complex with various agonists have identified D103^3.32^ and W264^6.48^ as critical residues for agonist binding through a combination of in vitro and *in silico* mutagenesis studies [27–30]. In all cases, alanine substitution at both positions significantly affected TAAR1 activation in response to agonists for both mouse and human receptors [27–31]. Notably, our dataset identified D103^3.32^N in WPR and SEAR, and W264^6.48^L in ROA and few others (Fig. 3B). In previous experimental studies, the negatively charged D103^3.32^ formed a critical salt bridge with the positively charged amine group of all complexed TAAR1 agonists [27–30, 32]. In comparison, the modified asparagine (D103^3.32^N) replaces the negative charge for a neutral charge, which was recently shown to abolish the binding PEA, tyramine, T1AM, and synthetic agonists including the atypical anti-psychotic asenapine [30]. Thus, D103^3.32^N will likely diminish binding of other agonists and raises a major concern regarding the efficacy of TAAR1 therapeutics in WPR and SEAR. Likewise, W264^6.48^, part of the CWxP motif, forms hydrophobic contacts with all complexed TAAR1 agonists [27–30]. Hence, the substitution of W264^6.48^L may influence the binding of selective TAAR1 agonists. Previous experimental studies show that W264^6.48^A completely inhibits TAAR1 activation by PEA and other agonists, with substitution to phenylalanine (W264^6.48^F) also reducing agonist potency [27–30]. This is likely divorced from the role of W264^6.48^ as a binding determinant and is more likely due to its role as part of the critical CWxP micro-switch [27] which is essential for helical rearrangements in response to ligand binding. In addition, alanine substitution of T194^5.42^ (found in SEAR) were previously experimentally validated and demonstrated to influence ligand induced activity of TAAR1 [27, 29, 30]. Hence it is plausible that populations belonging to these regions may see a varied response to TAAR1 therapeutics. In addition, these SNVs may contribute to symptomology associated with disease states such as attention deficit hyperactivity disorder (ADHD) due to their potential impact on endogenous ligand binding and trace amine activity [49].

Notably, previous mutagenesis studies demonstrated the importance of selective residues for ligand induced signalling. Our dataset showed SNVs at all these positions summarised in Fig. 5B. In this case, the alanine variants have been experimentally validated to affecting protein activity, including the common SNV T252^6.36^A, which affects G_αi_ coupling [29]. In addition, the G protein selectivity may be influenced by the variant V125^3.54^A (found in AR), to bias G-proteins towards a specific type (i.e. giving preference for G_αi_ over G_αs_) in a ligand dependent manner [29]. Such changes in G protein selectivity may reverse TAAR1s putative dopamine modulatory functions, possibly contributing to neuropsychiatric pathologies or affecting the ability of agonists to alleviate existing symptomology in African regions. Indeed, recent work suggests preferential activation of Gα_q_ pathways, or dual Gα_s_/Gα_q_ activation, by TAAR1 agonists has added benefit in animal models of schizophrenia [28]. While it remains to be seen if such observations translate to humans, such discoveries provide a framework for rational drug design but also highlight crucial evidence for how signalling SNVs may impact TAAR1 function and the clinical use of agonists for therapeutic benefit.

Whilst the clinical impact of TAAR1 SNVs affecting micro-switch motifs is yet to be elucidated, SNVs have been linked to pathologies in other GPCRs [50]. The DRY motif establishes intrahelical electrostatic interactions between D120^3.49^ and R121^3.50^ establishing a more critical interhelical ionic lock with TM6, both critical mechanisms for the receptor “packing”. These interactions may not be facilitated by residues with diverging electrostatic properties such as D120^3.49^G (ER), R121^3.50^S/C/L (variants S/C: common and variant L: AR) and Y122^3.51^C (SEAR/ER) [51–55]. In comparison, the interactions may still be established if the modified residue mirrors those electrostatic properties of original residues such as D120^3.49^E (SEAR) and R121^3.50^H (AR) [56, 57]. On the contrary, the structural features and stability facilitated by certain residues may be indispensable. The SEAR SNV P266^6.50^L of CWxP and P202^5.50^S of PIF motif (ROA and AR) may compromise the helical rearrangement facilitated by the cyclic proline. The helical rearrangement is critical for G protein coupling, thereby reducing the binding and signal transduction efficiencies [58]. Translationally, this implies regions harbouring TAAR1 SNVs may display varied response to agonists, warranting dose optimisation in certain populations belonging to certain geographical regions to achieve optimal pharmacological effects. Indeed, current literature shows a spectrum of receptor impact from micro-switch mutations. Some receptors are virtually unharmed, whilst others lose their functionality, warranting in vitro validation to assess the functional impacts of the SNVs predicted using *in silico* methods [51, 56, 57, 59]. Future studies may also consider the limitations surrounding *in silico*-based modalities and the absence of a population-specific categorisation. Datasets with such assortment can only be accomplished through targeted and controlled genomic studies, which may also mitigate population specific bias. Thus, further exploration of population specific SNVs is needed to aid in further development of personalised medicines.

In this study, we used NCBI’s dbGaP repository and the recent cryo-EM studies on human TAAR1 to identify, map and define the putative effect of SNVs on TAAR1 function. A total of 290 missense TAAR1 SNVs were found in our dataset. A structural mapping analysis of the primary data revealed over 40 SNVs that may influence TAAR1 activation by endogenous and exogenous ligands. Orthosteric SNV, D103^3.32^, found in certain regions (WPR, SEAR) and signalling SNVs (V125^3.54^A (AR) T252^6.36^A (common)) likely influence ligand recognition and alter signalling properties of TAAR1 ligands. From the current dataset, the highest number of TAAR1 SNVs were found in the AR, followed by SEAR, ROA, ER, and WPR, respectively, suggesting agents targeting TAAR1 may have differential therapeutic outcomes in geographically diverse populations, especially given most SNVs are predicted to reduce ligand binding. In essence, our study provides insights into the putative impact of missense SNVs on TAAR1 activation-signalling mechanisms and its implications with the use of next-generation therapeutics in population from various demographic regions. Functional validation and more rigorous in vitro and computational studies may help us better understand the clinical impact of SNVs. Future investigation of how TAAR1 SNVs affect expression and function, such as subcellular localisation, GPCR heterodimerisation, biased signalling, as well as analysis of SNVs outside of our primary dataset will build a molecular understanding of neuropsychiatric disorders and to the development of novel TAAR1 therapeutics.

### Electronic supplementary material

Below is the link to the electronic supplementary material.

Supplementary Material 1

## Data Availability

No datasets were generated or analysed during the current study.
