# Supplementary material for: Trace amine associated receptor 1: predicted effects of single nucleotide variants on structure-function in geographically diverse populations
Source: Hum Genomics. 2024 Jun 11;18:61. doi: 10.1186/s40246-024-00620-w (PMC11165750; doi:10.1186/s40246-024-00620-w)
Supplement: Supplementary file 1 — Supplementary Material 1 [file 40246_2024_620_MOESM1_ESM.docx]

**Trace amine associated receptor 1: Effects of single nucleotide variants on structure-function in geographically diverse populations**

Britto Shajan^1^, Shashikanth Marri^2^, Tarun Bastiampillai^3,4^, Karen J. Gregory^5,6^ , Shane D. Hellyer^5^ , Pramod C. Nair^1,2^

^1^Discipline of Clinical Pharmacology, College of Medicine and Public Health, Flinders University, Adelaide, SA, Australia

^2^Flinders Health and Medical Research Institute (FHMRI) College of Medicine and Public Health, Flinders University, Adelaide, SA, Australia

^3^Department of Psychiatry, Monash University, Parkville, Melbourne, VIC, Australia

^4^Discipline of Psychiatry, College of Medicine and Public Health, Flinders University, Adelaide, SA, Australia

^5^Drug Discovery Biology, Monash Institute of Pharmaceutical Sciences, Monash University, 381 Royal Parade, VIC 3052, Australia.

^6^ARC Centre for Cryo-electron Microscopy of Membrane Proteins, Monash Institute of Pharmaceutical Sciences, Monash University, Parkville, VIC, 3052, Australia

**Corresponding author:**

Dr Pramod Nair - Discipline of Clinical Pharmacology,

Flinders Medical Centre, College of Medicine and Public Health,

Flinders University, Adelaide, Australia.

Phone: 61-8-82043155

Email: [pramod.nair@flinders.edu.au](mailto:pramod.nair@flinders.edu.au)

| **rsID** | **SNV** |
| --- | --- |
| rs772197554 | L72^2.52^P |
| rs753020921 | S80^2.61^G |
| rs372879305 | H99^3.28^R |
| rs759733834 | D103^3.32^N |
| rs1777662264 | I104^3.33^S |
| rs774612586 | I104^3.33^V |
| rs1421620750 | S108^3.37^P |
| rs1562202086 | F112^3.41^L |
| rs1199836460 | V150^4.52^A |
| rs1235033813 | V150^4.52^I |
| rs774487833 | P151^4.53^A |
| rs763035708 | F154^4.56^L |
| rs1317154735 | S183^ECL2^F |
| rs530743674 | V184^ECL2^L |
| rs1360810948 | T194^5.42^A |
| rs772535179 | T197^5.45^I |
| rs772535179 | T197^5.45^S |
| rs748878284 | S198^5.46^Y |
| rs779799468 | W264^6.48^L |

**Supplementary table 1: Orthosteric SNVs identified from analysis of human TAAR1 cryo-EM structures.**

**Supplementary table 2: Microswitch SNVs identified in DRY, PIF, CWxP and NPxxY domains of human TAAR1.**

| **rsID** | **Microswitch** | **SNV** |
| --- | --- | --- |
| rs1275786770 | DRY | D120^3.49^E |
| rs1306244133 | DRY | D120^3.49^G |
| rs750088922 | DRY | R121^3.50^H |
| rs750088922 | DRY | R121^3.50^L |
| rs199758150 | DRY | R121^3.50^C |
| rs199758150 | DRY | R121^3.50^S |
| rs142991502 | DRY | Y122^3.51^C |
| rs1777653302 | PIF | P202^5.50^S |
| rs142169206 | CWxP | C263^6.47^R |
| rs142169206 | CWxP | C263^6.47^G |
| rs779799468 | CWxP | W264^6.48^L |
| rs778737689 | CWxP | P266^6.50^A |
| rs778737689 | CWxP | P266^6.50^S |
| rs1427520356 | CWxP | P266^6.50^L |
| rs748356783 | NPxxY | N300^7.49^S |
| rs147691560 | NPxxY | N300^7.49^K |


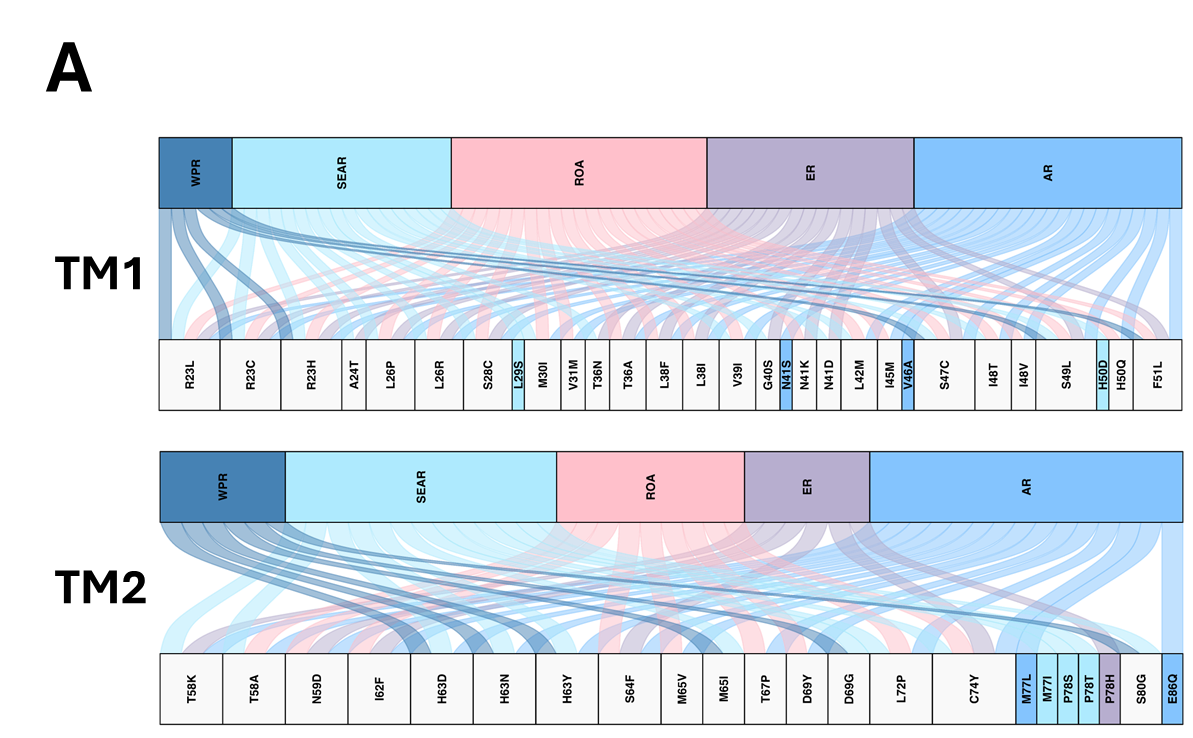


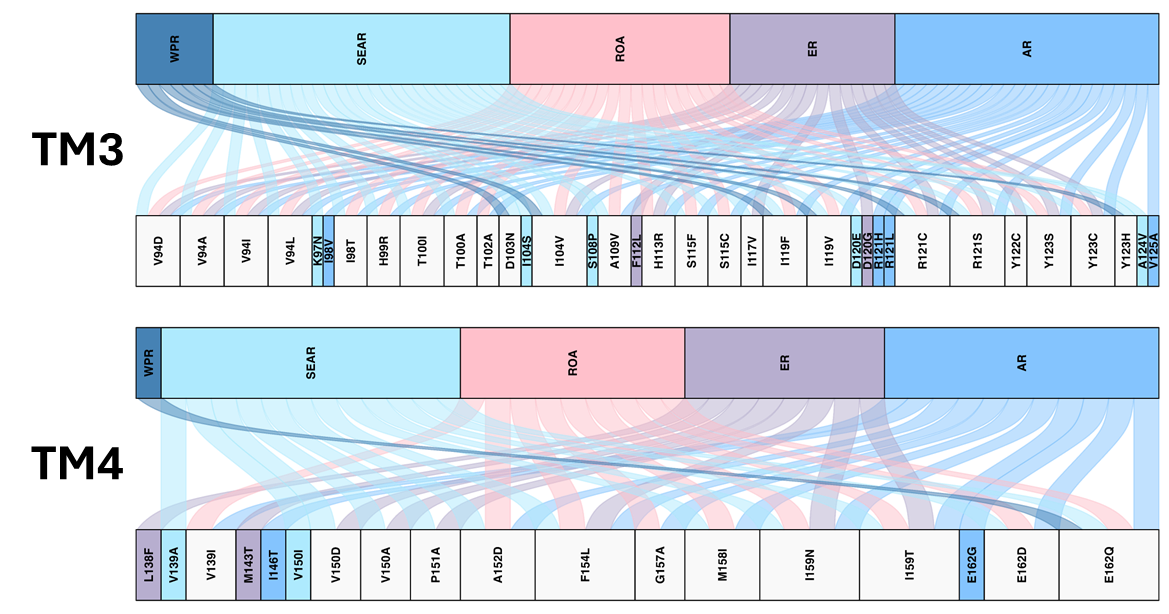


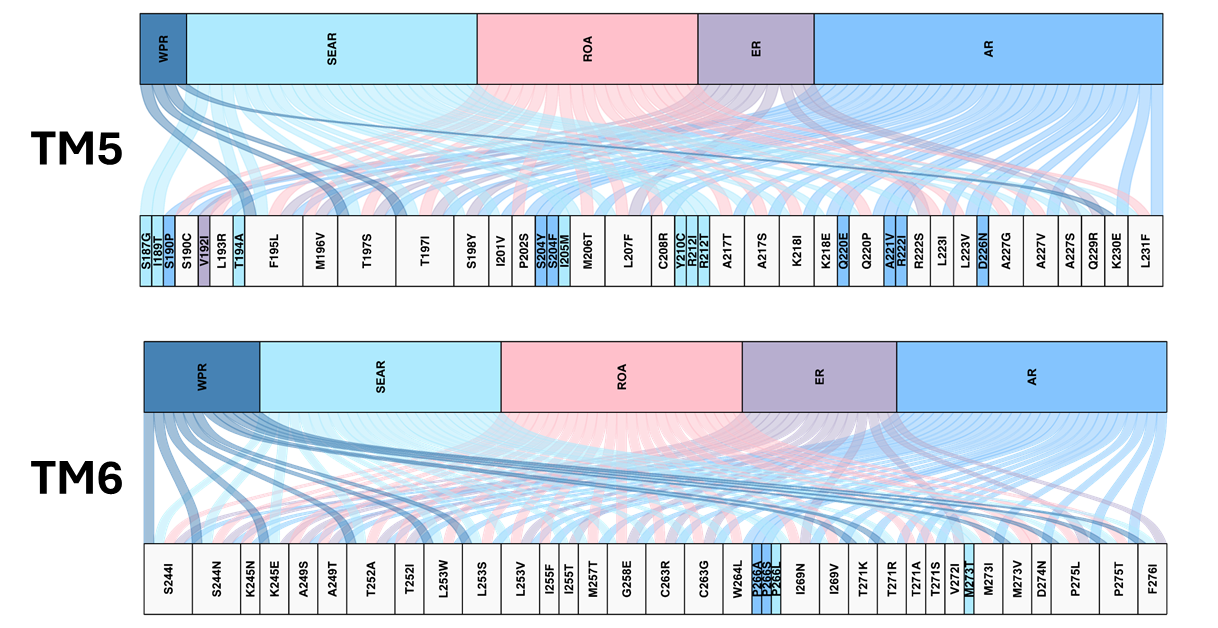


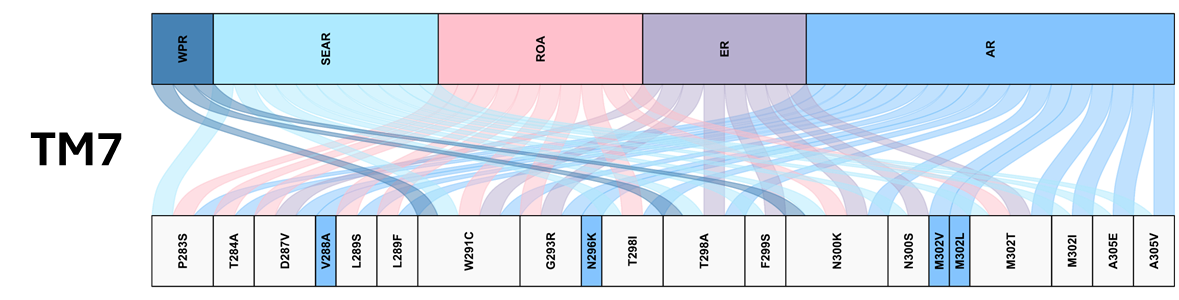


Supplementary figure 1: River plot demonstrating the demographic distribution of SNVs in TM 1-7. Top panel demonstrates the proportional burden of SNVs in WPR, AR, ROA, SEAR and ER. Bottom panels show the list of SNVs. The ribbons link each region with associated SNVs. Unique SNVs are colour matched with respective WHO region, and shared SNVs are coloured using light grey.


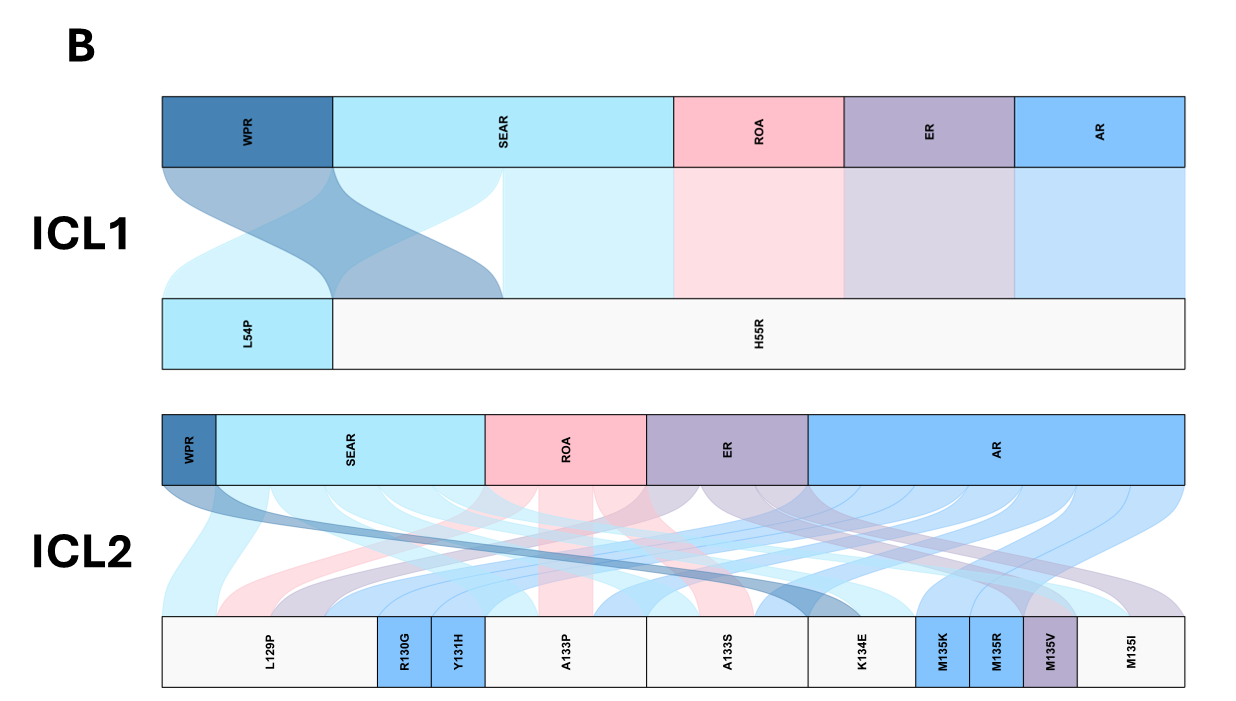


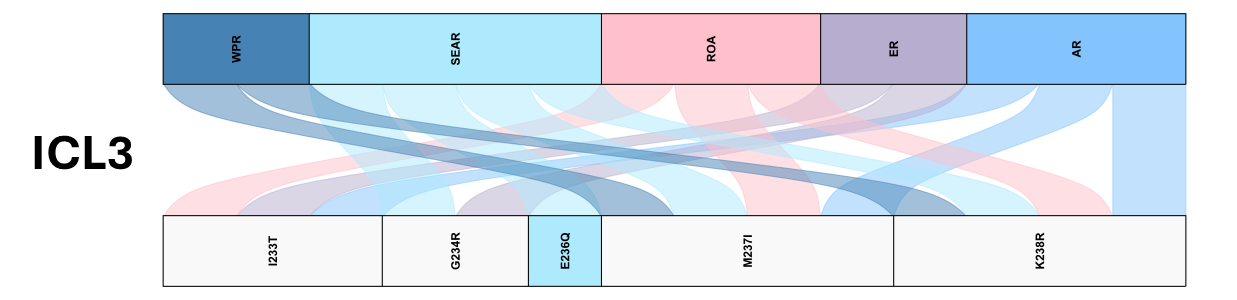


Supplementary figure 2: River plot demonstrating the demographic distribution of SNVs in ICL 1-3. Top panel demonstrates the proportional burden of SNVs in WPR, AR, ROA, SEAR and ER. Bottom panels show the list of SNVs. The ribbons link each region with associated SNVs. Unique SNVs are colour matched with respective WHO region, and shared SNVs are coloured using light grey.


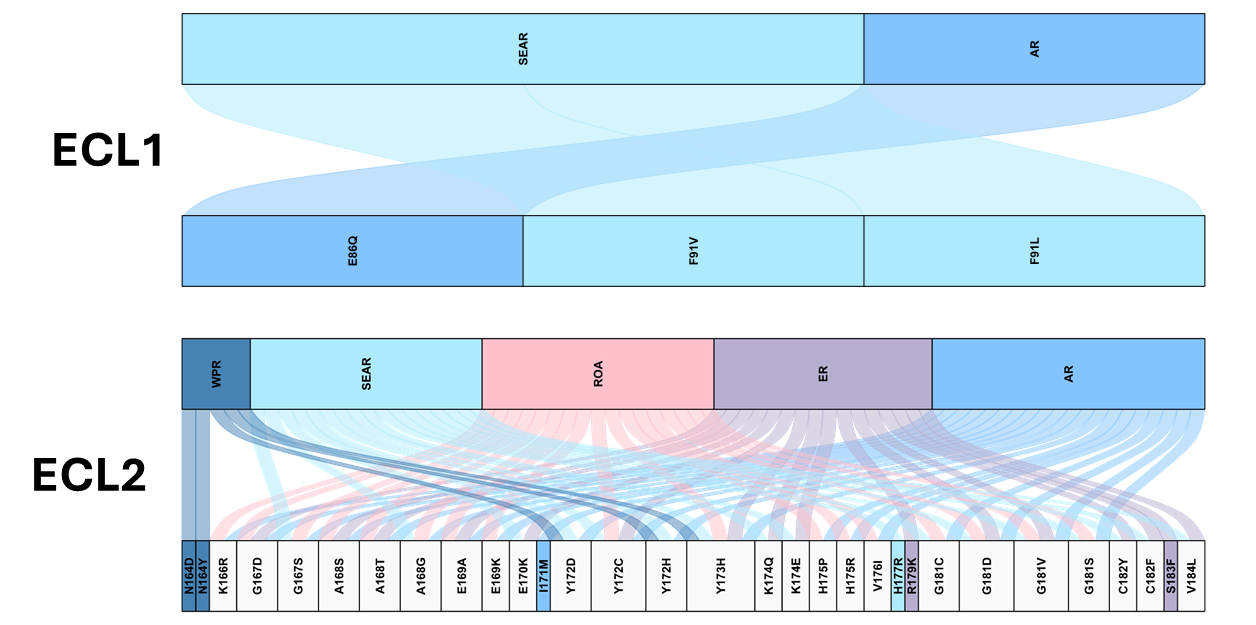


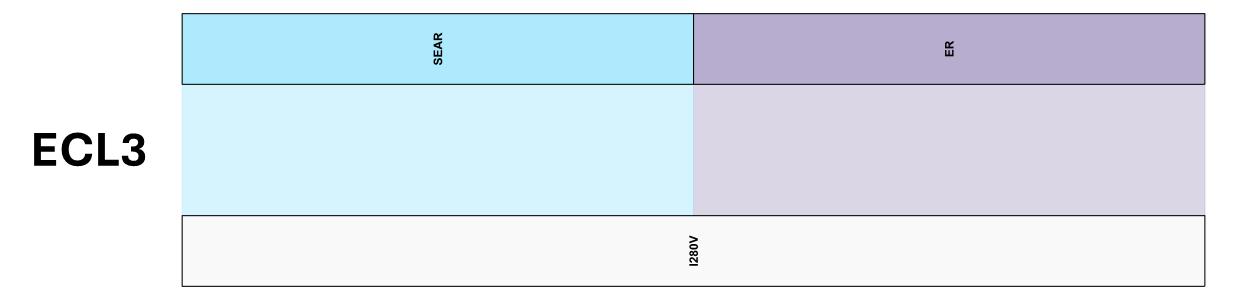


Supplementary figure 3: River plot demonstrating the demographic distribution of SNVs in ECL 1-3. Top panel demonstrates the proportional burden of SNVs in WPR, AR, ROA, SEAR and ER. Bottom panels show the list of SNVs. The ribbons link each region with associated SNVs. Unique SNVs are colour matched with respective WHO region, and shared SNVs are coloured using light grey.


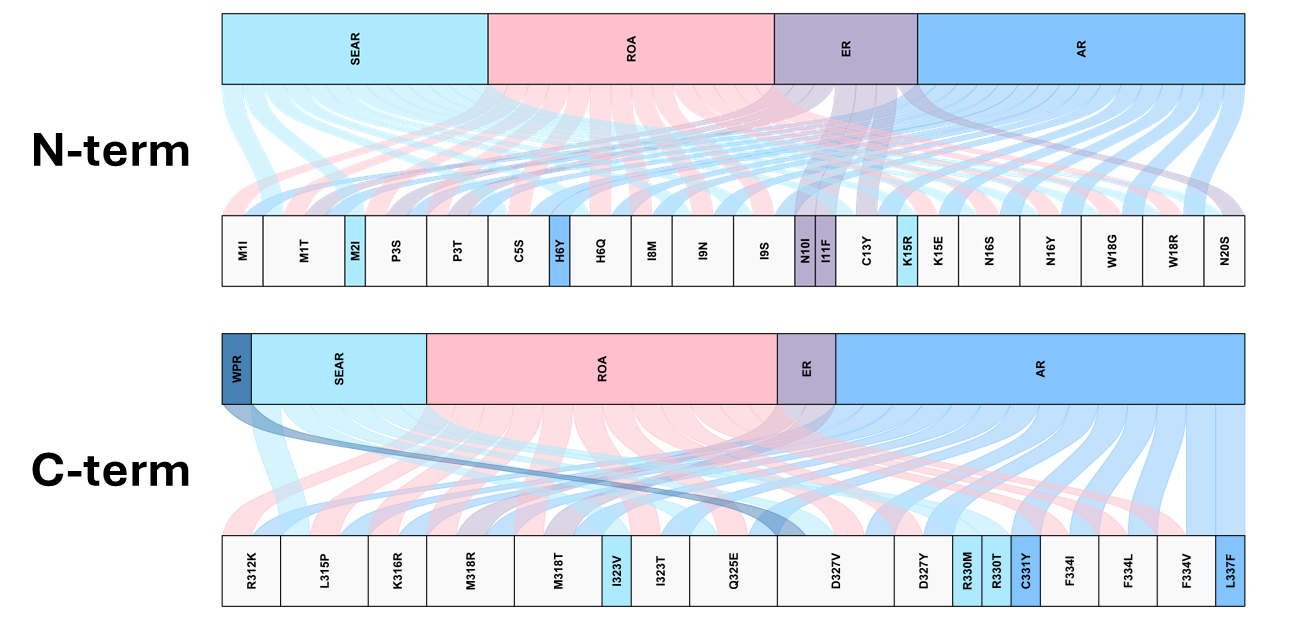


Supplementary figure 4: River plot demonstrating the demographic distribution of SNVs in N-term and C-term domains. Top panel demonstrates the proportional burden of SNVs in each region. Bottom panels list the SNVs. The ribbons link each region with associated SNVs. Unique SNVs are colour matched with respective WHO region, and shared SNVs are coloured using light grey.
